# Variations in Aspects of Neural Precursor Cell Neurogenesis in a Human Model of HSV-1 Infection

**DOI:** 10.1080/15476278.2022.2055354

**Published:** 2022-04-06

**Authors:** Wenxiao Zheng, Emily M. Benner, David C. Bloom, Vaishali Muralidaran, Jill K. Caldwell, Anuya Prabhudesai, Paolo A. Piazza, Joel Wood, Paul R. Kinchington, Vishwajit L. Nimgaonkar, Leonardo D’Aiuto

**Affiliations:** a Department of Psychiatry, Western Psychiatric Institute and Clinic, University of Pittsburgh School of Medicine, Pittsburgh, Pennsylvania, USA; b Second Xiangya Hospital, Xiangya School of Medicine, Central South University, Changsha, Hunan, China; c Department of Molecular Genetics & Microbiology, University of Florida College of Medicine, Gainesville, Florida, USA; d Department of Infectious Diseases and Microbiology, Pitt Graduate School Public Health, University of Pittsburgh, Pittsburgh, Pennsylvania, USA; e Department of Ophthalmology, University of Pittsburgh School of Medicine, Pittsburgh, Pennsylvania, USA; f Department of Molecular Microbiology and Genetics, University of Pittsburgh, Pittsburgh, Pennsylvania, USA

**Keywords:** Herpes simplex virus 1, human-induced pluripotent stem cells, neural stem cells, neural progenitor cells, acyclovir, brain organoids

## Abstract

Encephalitis, the most significant of the central nervous system (CNS) diseases caused by Herpes simplex virus 1 (HSV-1), may have long-term sequelae in survivors treated with acyclovir, the cause of which is unclear. HSV-1 exhibits a tropism toward neurogenic niches in CNS enriched with neural precursor cells (NPCs), which play a pivotal role in neurogenesis. NPCs are susceptible to HSV-1. There is a paucity of information regarding the influence of HSV-1 on neurogenesis in humans. We investigated HSV-1 infection of NPCs from two individuals. Our results show (i) HSV-1 impairs, to different extents, the proliferation, self-renewing, and, to an even greater extent, migration of NPCs from these two subjects; (ii) The protective effect of the gold-standard antiherpetic drug acyclovir (ACV) varies with viral dose and is incomplete. It is also subject to differences in terms of efficacy of the NPCs derived from these two individuals. These results suggest that the effects of HSV-1 may have on aspects of NPC neurogenesis may vary among individuals, even in the presence of acyclovir, and this may contribute to the heterogeneity of cognitive sequelae across encephalitis survivors. Further analysis of NPC cell lines from a larger number of individuals is warranted.

## Introduction

While HSV-1 is primarily associated with latent infection of peripheral nerve ganglia, it also exhibits a tropism toward the subventricular zone (SVZ) of the lateral ventricles and subgranular zone (SGZ) of the hippocampus.^1–4^ These regions are particularly enriched with neural stem cells/neural progenitor cells, which play a fundamental role in neurogenesis. We refer to neural stem cells and neural progenitor cells collectively as neural precursor cells (NPCs). Increasing evidence suggests that NPCs themselves are susceptible to HSV-1 in both *in vitro* and *in vivo* models.^5–8^ In general, susceptibility to HSV-1 infection depends on both viral (viral strain, route of infection, and viral dose) and host factors (cell type, strain, and age of the animal). It is not known whether specific cell types from different individuals exhibit comparable infection outcomes. The modeling of the interaction of HSV-1 with NPCs is particularly relevant in relation to the potential mechanistic link between HSV-1 infections and cognition deficits. There is a growing amount of evidence supporting the association of HSV-1 with cognition deficits^4, 9–12^. Furthermore, *in vivo* models of HSV-1 infections have shown that viral reactivation in CNS is followed by cognitive deficits.^13^ A recent study has provided evidence that these cognitive deficits are caused by impaired NPCs neurogenesis in the hippocampus.^14^

To the best of our knowledge, the effects of HSV-1 infection on neurogenesis are derived from animal models. The assessment of HSV-1 infections in neurons and NPCs of the species-relevant human host has been facilitated by differentiation of human induced pluripotent stem cells (hiPSCs), which provide an unprecedented opportunity to model the impact of HSV-1 on human neurogenesis.^8,15^

Here, we modeled the impact of HSV-1 infection, in the presence or absence of the gold standard antiviral ACV, on aspects of neurogenesis of NPCs derived from two separate individuals. Our results provide evidence of differences in the effect of the virus on the proliferation, self-renewal, and migration of NPCs generated from these two individuals. Also, we show variation in the efficacy of acyclovir to eliminate abnormalities in the aforementioned aspects of neurogenesis caused by HSV-1 infection in these two NPC lines.

## Materials and methods

### Virus strains

The following HSV-1 strains were employed in this study: KOS (VR-1493; ATCC), and a previously detailed KOS-based recombinant virus in which enhanced green fluorescent protein (EGFP) and monomeric red fluorescent protein (RFP) are reporters whose expression is driven by the viral promoters ICP0 and Glycoprotein C, respectively (HSV-1 DualFP).^8^

### Virus preparation

The virus stocks were prepared in the D’Aiuto laboratory at the University of Pittsburgh. 80–90% confluent monolayers of Vero cells were infected at a multiplicity of infection (MOI of 3 in DMEM medium supplemented with 2% FBS). After 2 hours the inoculum was removed, cells were washed and cultured for 2–3 days, until the appearance of full cytopathic effect (CPE). The cells were scaped and transferred along with the culture supernatant into 15 ml conical tubes. Cells were centrifuged at 1000 rpm for 5 minutes. The culture supernatant was removed, leaving behind 1.5 ml, and the cell pellet was resuspended using a vortex for 1–2 min. Cells were freeze-thawed three times. Debris was then removed by centrifuging at 3000 rpm for 5 minutes and the top culture supernatant containing cell-free viral particles was stored at −80°C until use. Virus titers were determined by the standard plaque assay as described below.

### HSV-1 plaque assay

Plaque assays were performed after confluent monolayers of Vero cells in 24-well tissue culture dishes were achieved. Once confluent, cells were infected with serial dilutions of HSV-1. After infection, supernatants were aspirated, cells were washed with PBS, and 1 ml of 3% (w/v) carboxymethyl cellulose (CMC) solution overlay medium was added. Plates were incubated under standard conditions (5% CO2, 37°C, and 100% humidity) for 72 hours. The CMC medium was then removed, cells were washed with PBS, and finally fixed in 4% formalin solution. After 1 h, the fixative was removed, and plaques were visualized by staining with gentian violet.

### Cell lines

Vero cells (CCL-81; ATCC) for plaque assay were maintained in Dulbecco’s modified Eagle’s medium (DMEM, Millipore Sigma D6171) supplemented with 10% fetal bovine serum (FBS, HyClone) and 5% antibiotic/antimycotic (Anti/Anti, Gibco 15,240–062).

Human-induced pluripotent stem cells (hiPSCs) were cultured in mTesR™ plus on Matrigel-coated tissue culture-treated plates (STEMCELL Technologies). The hiPSCs were established at the National Institute of Mental Health (NIMH) Center for Collaborative Studies of Mental Disorders-funded Rutgers University Cell and DNA Repository (RUCDR) (http://www.rucdr.org/mental-health). The control steps included the analysis of pluripotency markers NANOG, Oct4, TRA60, TRA811, OSX2 and SSEA4. We subsequently conducted karyotyping, array comparative genomic hybridization (aCGH) assays and short tandem repeat (STR) profiling and compared them with donor genomic DNA to evaluate structural changes in genomic DNA during the generation of hiPSC lines.

**Figure 1.**
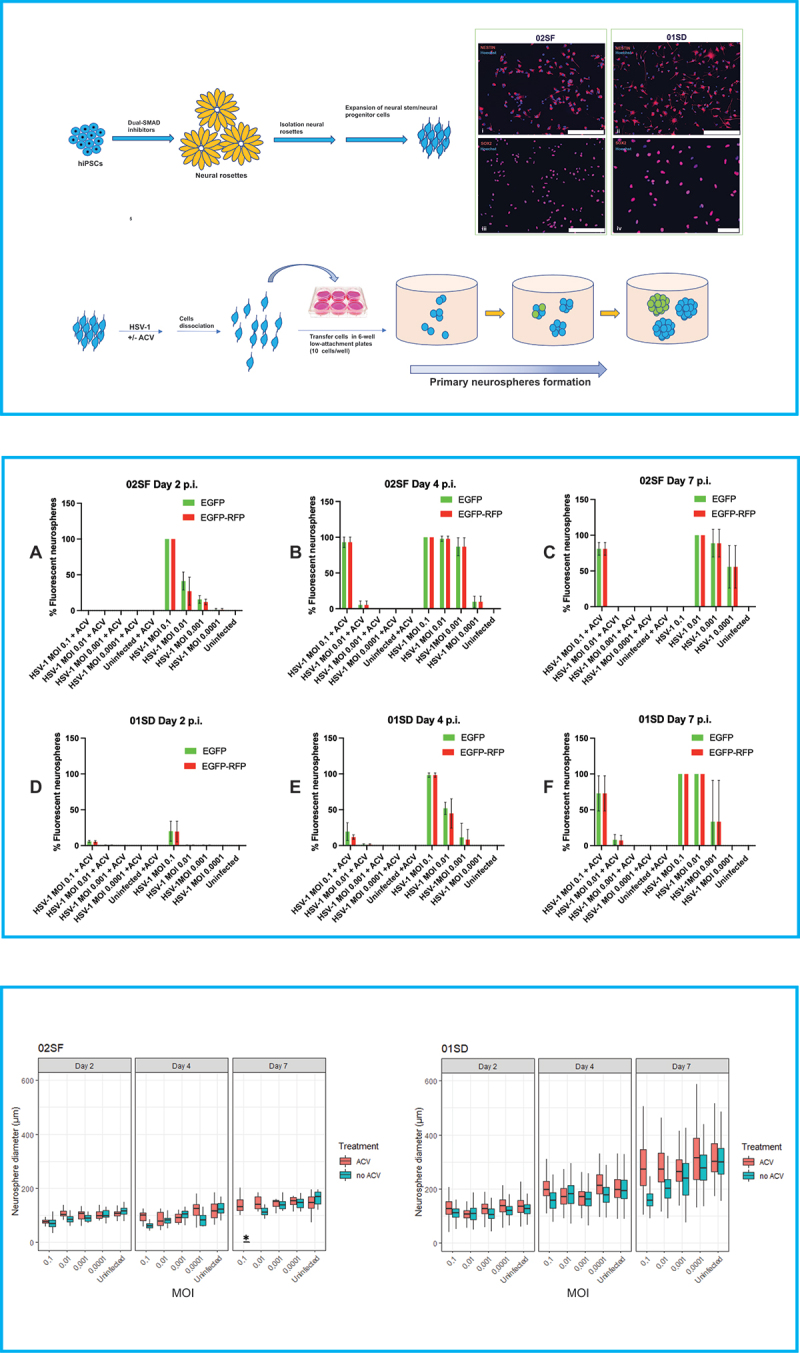
Variability of the effect of HSV-1 on the proliferative activity of NPCs. Top panel – upper left: Schematic flow diagram to depict the stages of differentiation of hiPSCs into NPCs. Top panel – upper right: Microphotographs depicting the expression of the NPC markers NESTIN and SOX2 in 02SF and 01SD. Scale bar: 250 microns in (i–iii) and 75 microns in (iv). Top panel – Bottom: Schematic diagram of the neurosphere formation assay. 02SF and 01SD NPCs were infected with HSV-1 expressing the EGFP and RFP reporter genes under the control of the viral promoter genes ICP0 and gC, respectively, at MOIs 0.1–0.0001 in the presence or absence of Acyclovir. After 1 hour the inoculum was removed, infected and uninfected monolayer cultures were washed and dissociated to single cells. Cell suspensions were transferred into low attachment plates to allow for the neurospheres (primary neurospheres) formation. Middle panel: Proportion of neurospheres derived from infected 02SF and 01SD NPCs exhibiting EGFP^+^ and EGFP^+^-RFP^+^ cells at days 2 (a, d), 4 (b, e), and 7 p. I (c, f). Bottom panel: Comparison of the diameter of primary neurospheres derived from uninfected and infected neurospheres at the aforementioned time points. The experiments were performed in triplicate for both lines. Each biological replicate at day 2 p.i. contained at least 40 neurospheres in 02SF cultures and 50 in 01SD cultures. Error bars are standard deviations. The asterisk symbol indicates the cultures where the neurospheres that lost their structural integrity.

Two human NPC lines were employed in this study. They were derived from hiPSC lines 73–56010-01 (denoted 01SD) and 73–56010-02 (denoted 02SF), as follows: hiPSCs were cultured in mTeSR1-plus medium supplemented with dual SMAD inhibitors SB 431542 and LDN 193189 to promote neural induction. After 8–10 days, neural rosettes were manually isolated, transferred into Matrigel coated plates and cultured in StemDiff Neural Progenitor Medium (STEMCELL Technologies) for the expansion of NPCs. The expression of the NPCs markers NESTIN and SOX2 was analyzed (Figure 1, *top panel*).^16^ All cells were cultured in standard conditions (37°C, 5% CO2, and 100% humidity).

Alt Text The top panel shows a schematic flow diagram that depicts the differentiation of neuronal progenitor cells from induced pluripotent stem cells and their characterization by immunohistochemistry. It also shows the generation of neurospheres from 02SF and 01SD neuronal progenitor cells infected with an HSV-1 construct expressing the EGFP and RFP reporter genes under the control of the viral promoter genes ICP0 and gC, respectively, at MOIs 0.1–0.001 in the presence or absence of acyclovir. The middle panel shows the proportion of neurospheres derived from infected 02SF and 01SD NPCs exhibiting EGFP+ and EGFP+-RFP+ cells at days 2, 4, and 7 p. The bottom panel shows the comparison of the diameter of primary neurospheres derived from uninfected and infected neurospheres at the aforementioned time points.

### Neurosphere formation assay

Generation of primary neurospheres and HSV-1 infection. NPCs were seeded in Matrigel-coated 12-well plates at the density of 1 × 10^5^ cells/well and cultured in StemDiff™ Neural progenitor (NP) medium (STEMCELL Technologies) until cells were approximately 80–85% confluent. NPCs were infected with HSV-1 dualFP virus at MOIs 0.0001, 0.001, 0.01, and 0.1 in the presence or the absence of acyclovir (50 µM) and cultured in NP medium. NPCs that were infected in the presence of ACV were also pretreated with the antiviral for 24 hours. All conditions were performed in triplicates. One hour after the infection, inoculum was removed, and cells were washed with PBS. All NPCs were dissociated manually and seeded into low attachment 6-well plates at a density of approximately 1 × 10^5^ cells per well to allow for the formation of neurospheres. The intensity of the fluorescence in the neurospheres was observed under the fluorescent microscope on a daily basis. The number and the size of spheres on days 2, 4 and 7 post infection were measured as follows: Different microphotographs were taken in each culture well to capture all of the neurospheres. Next, the images were compiled. The total number of neurospheres, as well as the number of EGFP^+^ and EGFP^+^-RFP^+^ neurospheres, was then counted manually. The diameter of each was measured using ImageJ.

Generation of secondary neurospheres. On day 7 post infection (day 7 p.i.), primary neurospheres from different conditions were gathered, incubated with Accutase (BioLegend) for 10 minutes and dissociated into single-cell suspension by manually pipetting. Cells were transferred to new six-well low attachment plates in NP medium, with or without the presence of ACV. The number, size, and intensity of fluorescence of the secondary neurospheres were analyzed at days 2, 4, and 7 p.i.

### Migration assay

To assess NPCs migration, NPCs propagated in 12-well Matrigel-coated plates were infected at MOIs 0.0001, 0.001, 0.01, and 0.1 in the presence or in the absence of ACV (50 µM) and cultured in NP medium. One-hour p.i. the inoculum was removed, cells were rinsed with PBS once, and all uninfected and infected NPCs were dissociated manually and transferred at a density of 2 × 10^4^ cells per well into low attachment round (U)-bottom 96-well plates to form neurospheres (one in each well) with comparable sizes. Seventy-two hours later, the neurospheres generated were transferred into Matrigel-coated 48-well plates with one sphere per well. After 48 hours, the average migration of NPCs was assessed by measuring the distance of the five furthest cells from the edge of each neurosphere (greatest cellular migration) using Image J.^17^ A minimum of 10 neurospheres per condition were analyzed.

### Cell proliferation assay

The cell proliferation of NPCs was measured by Click-&-Go EdU Proliferation Assay Kit (Click Chemistry Tools) assay, according to the manufacturer’s protocol. NPCs were seeded in 24-well plates at a density of 10^5^cells/well. Three biological replicates were generated for each NPC line. After 24 hours, cells were treated with 10 μM of 5-ethynyl-2′-deoxyuridine (EdU) for 1.5 h at 37°C, fixed with 4% Paraformaldehyde in PBS for 5 min at room temperature, washed twice with 3% BSA in PBS, and washed with 0.5% Triton X-100 in PBS and incubated at room temperature for 20 minutes. The cells were then treated with a reaction cocktail made with 1x Reaction Buffer, Copper(II) Sulfate, Azide Solution, and 1x Reducing Agent. The cells were incubated at room temperature away from the light for 30 minutes with 300 μL of the reaction cocktail. Cells were then washed again with 3% BSA in PBS followed by a rinse with Wash Buffer. The cells were then stained with 1 ml of Hoechst 33,342 solution (5 μg/ml) and incubated for 30 minutes protected from light. Cells that incorporated EdU were analyzed using a fluorescent microscope (Leica CTR5500). Four random fields for each biological replicate were analyzed using ImageJ software 2.0.0-rc-43/1.50e.

### Statistical analysis

The effect of HSV-1 on the proliferation of NPCs was assessed by measuring the diameters of neurospheres in two cell lines at three time points, at four MOIs plus uninfected controls, and in the presence or absence of acyclovir. We also measured the concurrent percentage of EGFP^+^ and EGFP^+^-RFP^+^ neurospheres (both fluorescent reporter genes were used as a proxy for viral gene expression to compare the kinetics of viral transcription in both infected NPC lines). Secondary neurosphere generation from cells gleaned from the previous experiment was observationally assessed regarding their ability to form new spheres. Migration of NPCs from neurospheres was assessed using a standard distance measurement procedure, with the outcome being mean distanced traveled by a sample of cells. We also assessed EGFP^+^-RFP^+^ fluorescence in these spheres and cells.

A one-way analysis of variances (ANOVA) with Bonferroni corrected post-hoc tests was utilized for the analysis of neurosphere diameters across days in 02SF and 01SD cell lines, at four different MOIs and uninfected controls, both treated with ACV and untreated. Student’s t-tests were conducted to compare results between specific pairs of cell lines and conditions in the other experiments. Statistical analyses were performed using GraphPad Prism 9.1.2 (225).

## Results

### Effect of HSV-1 on the proliferation of two genetically related NPC lines

The neurosphere formation assay represents an invaluable *in vitro* model to investigate the proliferation of NPCs (see Materials and Methods).^18,19^ The neurospheres diameter reflects the proliferative activity of NPCs.^19^ A schematic representation of the neurospheres formation assay is depicted in Figure 1 (top panel). NPCs generated from 02SF and 01SD hiPSC lines were infected with HSV-1 DualFP at MOIs 0.1–0.0001 in the presence or the absence of ACV as described in the Materials and Methods section. Infected and uninfected cells were dissociated and transferred into low attachment 6-well plates at a density of 1 × 10^5^ cells/well for the formation of neurospheres, which were analyzed at days 2, 4, and 7.

### Primary neurospheres in cultures unexposed to ACV at day 2 post infection

Differences in the proportion of fluorescent neurospheres between 02SF and 01SD were observed starting from day 2 p.i. At this time point, all neurospheres generated from 02SF NPCs, which were acutely infected at an MOI 0.1 expressed both fluorescent reporter genes. The proportion of fluorescent neurospheres decreased at lower MOIs (Figure 1, *middle panel*, A; Table 1). The diameters of infected neurospheres increased significantly as the MOI decreased from 0.1 to 0.0001, based on Student's t-test (p values for all comparisons <0.01) (Figure 1, *bottom panel*). Furthermore, at all MOIs the diameters of 02SF neurospheres were significantly smaller than uninfected neurospheres (p < .0160) (Figure 1, *bottom panel*), indicating that HSV-1 affects the 02SF NPCs proliferation in a viral dose-dependent fashion.

**Table 1.** Percentages (%) of fluorescent primary neurospheres at different time points

		Day 2 p.i.					Day 4 p.i.					Day 7 p.i.				
02SF																
	MOI	0.1	0.01	0.001	0.0001	0	0.1	0.01	0.001	0.0001	0	0.1	0.01	0.001	0.0001	0
ACV (-)	EGFP^+^	100	41.3	15.6	1.09	0	100	97.86	86.76	9.64	0	Degenerated	100	88.86	55.79	0
	EGFP^+^- RFP^+^	100	27.1	11.9	1.09	0	100	97.86	86.76	9.64	0	Degenerated	100	88.86	55.79	0
ACV (+)	EGFP^+^	0	0	0	0	0	93	5.33	0	0	0	80.88	0	0	0	0
	EGFP^+^- RFP^+^	0	0	0	0	0	93	5.33	0	0	0	80.88	0	0	0	0
01SD																
	MOI	0.1	0.01	0.001	0.0001	0	0.1	0.01	0.001	0.0001	0	0.1	0.01	0.001	0.0001	0
ACV (-)	EGFP^+^	20.06	0.74	0.28	0	0	98.25	51.23	11.33	0	0	100	100	33.3	0	0
	EGFP^+^- RFP^+^	19.5	0.74	0.28	0	0	98.25	44.76	8.22	0	0	100	100	33.3	0	0
ACV (+)	EGFP^+^	5.76	0.33	0	0	0	19.43	1.23	0	0	0	72.89	8.1	0	0	0
	EGFP^+^- RFP^+^	5.26	0.33	0	0	0	11.86	1.23	0	0	0	72.89	7.22	0	0	0

*MOI: Multiplicity of infection; EGFP: Enhanced green fluorescent protein; RFP: Red fluorescent protein; p.i.: post infection.

The proportion of fluorescent 01SD neurospheres in the same infection conditions at day 2 p.i. was reduced relative to 02SF across all MOIs, with approximately 20% of fluorescent neurospheres observed in cultures infected at MOI 0.1 (Figure 1, *middle panel*, D; Table 1). At MOIs 0.01–0.001 the proportion of fluorescent neurospheres was 0.74% and 0.28%, respectively (Table 1). At an MOI of 0.0001, no fluorescent cells were detected in neurospheres (Figure 1, *middle panel*, D; Table 1). Thus, over a range of MOIs, the proportion of fluorescent neurospheres was considerably lower for 01SD than 02SF, indicating different transcriptional activity of HSV-1 in these two related hiPSC-derived NPC lines following infection with HSV-1. Despite this lower level of HSV-1 transcription, at day 2 p.i., a significant difference in diameters of the 01SD neurospheres generated at all MOIs 0.1–0.001 was observed when compared to those uninfected (uninfected vs MOIs 0.1–0.001: p < .0001; uninfected vs MOI 0.0001: p < .0356; Figure 1, *bottom panel*).

However, 02SF neurospheres infected at all MOIs, including at MOI 0.0001, were significantly smaller than those from uninfected NPCs based on Student's t-test (p < .007) (Figure 1, *bottom panel*). Together, these results indicate that HSV-1 affects the NPCs proliferation more markedly in 02SF than in the 01SD line.

### Primary neurospheres in cultures treated with ACV at day 2 post infection

In ACV-treated 02SF cultures, no fluorescent neurospheres were observed at day 2 p.i., while ACV-treated HSV-1 infected 01SD cultures showed a small fraction of fluorescent neurospheres only at MOIs of 0.1 and 0.01 (Figure 1, middle panel, A, D; Table 1). In addition, in the presence of ACV, the size of the infected 02SF neurospheres was comparable to the uninfected neurospheres, except for those at an MOI of 0.1 (Student's t-test, p < .0001). However, infected 01SD neurospheres were smaller than the uninfected neurospheres at all MOIs except 0.0001 (p = .009) (Figure 1, *bottom panel*). These results indicate higher efficacy of ACV in reducing abnormalities of NPCs proliferation in 02SF than 01SD, however it did not fully protect cells in either line.

### Primary neurospheres in cultures unexposed to ACV at day 4 post infection

On day 4 p.i., the proportion of 02SF neurospheres containing EGFP^+^ and EGFP^+^-RFP^+^ cells generated from NPCs infected at MOIs of 0.01–0.0001 increased substantially as compared to day 2 (Table 1), while the proportion of fluorescent neurospheres generated from acutely infected 01SD NPCs increased to a much lesser extent (Table 1), with no fluorescent 01SD neurospheres observed at an MOI of 0.0001 (Table 1; Figure 1, middle panel, B, E). 01SD neurospheres significantly increased their diameters at all MOIs (p < .0001), while a significant increase in 02SF neurospheres was observed only at MOI 0.0001 (p = .001). However, the diameter of the neurospheres in the infected conditions was significantly reduced when compared to those uninfected (02SF: p < .001, 01SD: p < .0001).

### Primary neurospheres in cultures treated with ACV at day 4 post infection

At day 2 p.i., no fluorescent 02SF neurospheres were observed in ACV-treated cultures. However, at day 4 p.i. approximately 93% of 02SF neurospheres exposed to ACV were EGFP^+^ and EGFP^+^-RFP^+^ at an MOI of 0.1. In 01SD cultures exposed to ACV, a modest increase in the proportion of EGFP^+^ and EGFP^+^-RFP^+^ neurospheres was observed at an MOI of 0.1 (from 5.76% and 5.26% to 19.43 and 11.86, respectively). A small proportion (<2%) of fluorescent 02SF and 01SD neurospheres was observed at an MOI of 0.01 in the presence of ACV (Table 1). At MOIs of 0.001–0.0001 no fluorescent neurospheres were detected in 02SF and 01SD in ACV-treated cultures (Figure 1, *middle panel*, B, E; Table 1).

An increase in diameters of ACV-treated infected 02SF neurospheres was observed only at MOI 0.0001 as compared to day 2 (Figure 1, *bottom panel*; Table 2), while the diameter of 01SD neurospheres increased significantly at all MOIs (Student's t-test, p < .0001) (Figure 1, *bottom panel*; Table 2). The diameter of infected 02SF was significantly smaller at all MOIs as compared to uninfected neurospheres (P < .0001). The infected 01SD neurospheres at MOIs 0.01 and 0.001 were significantly smaller than those uninfected (p < .0001). However, we did not detect significant differences at MOI 0.0001 or, unexpectedly, at MOI 0.1 as compared to those uninfected (Figure 1, *bottom panel*).

**Table 2.** Statistical significance of comparison of diameters of 02SF primary

02SF						
	MOI	0.1	0.01	0.001	0.0001	0
	**ANOVA**	F(2,78) = 101.5, p < .0001	F(2,58) = 7.1, p = .002	F(2, 55) = 26.1, p < .0001	F(2,55) = 36.9, p < .0001	F(2,47) = 18.3, p < .0001
**ACV(-)**	**Day4 vs Day2**	NS	NS	NS	0.01	NS
	**Day7 vs Day2**	<0.0001	0.005	<0.0001	<0.0001	<0.0001
	MOI	0.1	0.01	0.001	0.0001	0
	**ANOVA**	F(2,60) = 32.6, p < .0001	F(2, 50) = 16.8, p < .0001	F(2,54) = 15.1, p < .0001	F(2,56) = 19.5, p < .0001	F(2,54) = 9.1, p = .0004
**ACV(+)**	**Day4 vs Day2**	NS	NS	NS	0.036	NS
	**Day7 vs Day2**	<0.0001	0.0005	0.0005	<0.0001	0.0003
**Neuroshperes**						
**01SD**						
	MOI	0.1	0.01	0.001	0.0001	0
	**ANOVA**	F(2, 490) = 96.0, p < .0001	F(2,416) = 183.4, p < .0001	F(2,520) = 315.4, p < .0001	F(2, 505) = 527.9, p < .0001	F(2, 484) = 435.8, p < .0001
**ACV(-)**	**Day4 vs Day2**	<0.0001	<0.0001	<0.0001	<0.0001	<0.0001
	**Day7 vs Day2**	<0.0001	<0.0001	<0.0001	<0.0001	<0.0001
	MOI	0.1	0.01	0.001	0.0001	0
	**ANOVA**	F(2,483) = 318.5, p < .0001	F(2, 512) = 407.2, P < .0001	F(2,492) = 415.1, P < .0001	F(2, 365) = 210.5, P < .0001	F(2, 424) = 311.4, P < .0001
**ACV(+)**	**Day4 vs Day2**	<0.0001	<0.0001	<0.0001	<0.0001	<0.0001
	**Day7 vs Day2**	<0.0001	<0.0001	<0.0001	<0.0001	<0.0001

*One-way analyses of variance were conducted, and p values shown in the table were adjusted after Bonferroni’s multiple comparison test, p = 0.05 was considered statistically significant.

*MOI: Multiplicity of infection*

### Primary neurospheres in cultures unexposed to ACV at day 7 post infection

All the neurospheres derived from 02SF NPCs infected at an MOI of 0.1 exhibited morphological changes and appear to have lost their structural integrity at day 7 p.i. (Supplementary Figure 1). In contrast, 01SD neurospheres derived from NPCs infected at the same MOI were still intact (Supplementary Figure 1), although, in total, they expressed both fluorescent reporters (Figure 1, *middle panel*). At an MOI of 0.01 all the 02SF and 01SD neurospheres were EGFP^+^-RFP^+^. The main difference between the two NPC lines was that a substantial portion of 02SF neurospheres were losing their structural integrity and appeared to show overt cytopathic effects, while 01SD neurospheres were still intact and only showed minimal signs of cytopathology (Supplementary Figure 1). At an MOI of 0.001, the average proportion of fluorescent 02SF neurospheres was higher than in 01SD (Table 1). Also, a proportion of 02SF neurospheres were disassembled (Supplementary Figure 1). At MOI 0.0001, fluorescent neurospheres were detected only in 02SF but not in 01SD (Figure 1, *middle panel*, C, F; Table 1). The diameters of the 02SF and 01SD neurospheres were larger at day 7 at all MOIs as compared to day 4 (Figure 1, *bottom panel*, Table 2). However, as stated above, the structural integrity of a fraction of 02SF neurospheres in the absence of ACV was compromised.

### Primary neurospheres in cultures treated with ACV at day 7 post infection

In the presence of ACV, a modest reduction in the percentage of fluorescent 02SF neurospheres at MOIs 0.1–0.01 was observed at day 7 p.i. (Figure 1, *middle panel*, C; Table 1). Furthermore, the size of infected 02SF neurospheres at different MOIs was comparable to uninfected neurospheres (Figure 1, *bottom panel*). Conversely, at this time point, in the presence of ACV, a 3.75-fold and 6.5-fold increase in the percentage of EGFP^+^ and EGFP^+^-RFP^+^ 01SD neurospheres (compared to day 4 p.i.), respectively, was observed at MOI 0.1 and a 6.14-fold and 5.85-fold increase of EGFP^+^ and EGFP^+^-RFP^+^ neurospheres, respectively, at MOI 0.01 (Figure 1, *middle panel*, F; Table 1). The diameters of the 01SD neurospheres at MOIs 0.1, 0.01, and 0.001 were significantly reduced when compared to uninfected neurospheres (p = .0011, p = .0002, and p < .0001, respectively), while no significant difference was observed only at MOI 0.0001. Overall, the diameters of 02SF and 01SD increased significantly when compared to day 2 p.i (Figure 1, *bottom panel;* Table 2). Together, these observations highlight the disparities in the effectiveness of ACV in separately derived lines.

Considering the larger diameter of 01SD neurospheres when compared to 02SF the difference in proliferative activity between 01SD and 02SF NPCs was analyzed. The proliferation of both NPC lines, analyzed by Click-&-Go EdU assay, showed a significant reduction (p = .00146) of the thymidine nucleoside analog EdU incorporation in 02SF NPCs when compared to 01SD NPCs (46.6% EdU^+^ cells in 02SF vs 59.6% EdU^+^ cells in 01SD) (Supplementary Figure 2), thus confirming the higher proliferation rate of 01SD NPCs.

### Secondary neurospheres: Day 7 post dissociation

**Figure 2.**
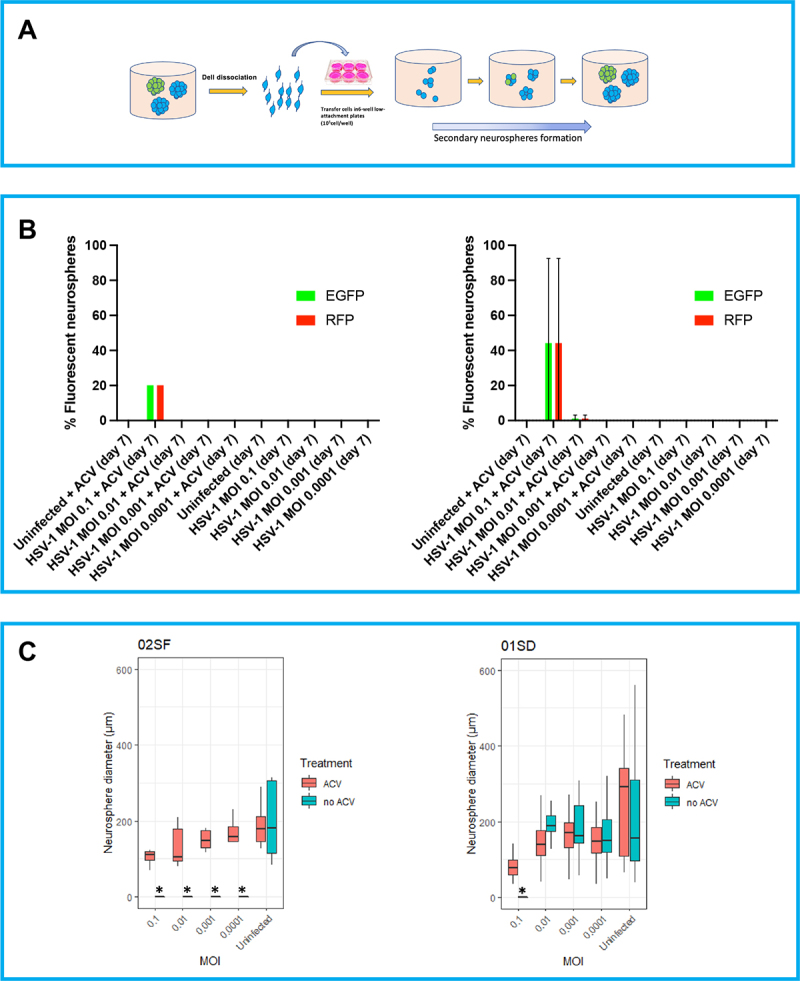
Variability in the ability of HSV-1 infected NPCs to retain the self-renewal capacity. A: Schematic diagram of the neurosphere formation assay. At day 7 p.i. primary neurospheres derived from uninfected and infected 02SF and 01SD NPCs were dissociated, and single cell NPCs were transferred into low attachment plates to allow for the formation of secondary neurospheres. B: Proportion of the fluorescent neurospheres at day 7. **C**: Comparison of the diameter of secondary 02SF and 01SD neurospheres. Error bars are standard deviations.

To investigate the self-renewal capacity of NPCs after infection with HSV-1, the 02SF and 01SD neurospheres at day 7 p.i. (primary neurospheres) were dissociated, transferred into low attachment 6-well plates, and re-cultured for a further 7 days (Figure 2a). The proportion of cells in the neurospheres generated from 01SD NPCs infected at MOIs of 0.01–0.0001, in the absence of ACV, retained the ability to form secondary neurospheres, while 02SF NPCs from infected cultures not treated with ACV were unable to form secondary neurospheres, suggesting that HSV-1 infection impaired 02SF NPCs self-renewal capacity (Figure 2c). In the presence of ACV, 02SF and 01SD secondary neurospheres were generated at all MOIs; EGFP^+^-RFP^+^ cells were detected only at an MOI of 0.1 (Figure 2b).

Alt Text The panel A shows a schematic diagram for the generation of secondary neurospheres formation from uninfected and infected primary neurospheres. The panel B shows the proportion of the fluorescent secondary neurospheres at day 7. The panel C shows a comparison of the diameters of secondary 02SF and 01SD neurospheres.

In summary, these data show that HSV-1 affected the ability of NPCs to proliferate, depending on viral dose (Figure 1, *bottom panel*). The expression of the fluorescent reporter genes EGFP and RFP as proxy of viral gene expression indicates that the kinetics of viral transcription was slower in 01SD than 02SF (Figure 1, *middle panel*). The ACV treatment eliminated the inhibitory effect of HSV-1 on NPCs proliferation in 02SF cultures but not in 01SD cultures infected at MOIs of 0.1–0.001 (Figure 1, *bottom panel*). It is clear that the infection, particularly at higher MOI, reduced primary neurospheres expansions, but that 01SD was able to expand further under the same infection conditions as used for 01SD. This evidence suggests that the increased proliferation rate of 01SD NPCs, when compared to 02SF, may in part, compensate for the reduced efficacy of ACV in preventing the effect of viral infection on NPCs proliferation. Self-renewing NPCs were present in 01SD neurospheres generated from NPCs acutely infected at MOIs of 0.01–0.0001 but not in 02SF NPCs (Figure 2). Neurospheres generated from 01SD NPCs infected at an MOI of 0.1 displayed more normal morphology and cytopathology than the ones generated from infected 02SF. In fact, on day 7 p.i. at an MOI of 0.1, no intact 02SF neurospheres in the condition MOI 0.1 were observed. At MOIs of 0.01 and 0.001 both intact and fragmented 02SF neurospheres were observed. Conversely, 01SD neurospheres generated from acutely infected NPCs did not show disaggregation under any conditions (Figure 1, *top panel*; Supplementary Figure 1).

### HSV-1 differentially impairs the extent the migration of NPCs from different individuals

**Figure 3.**
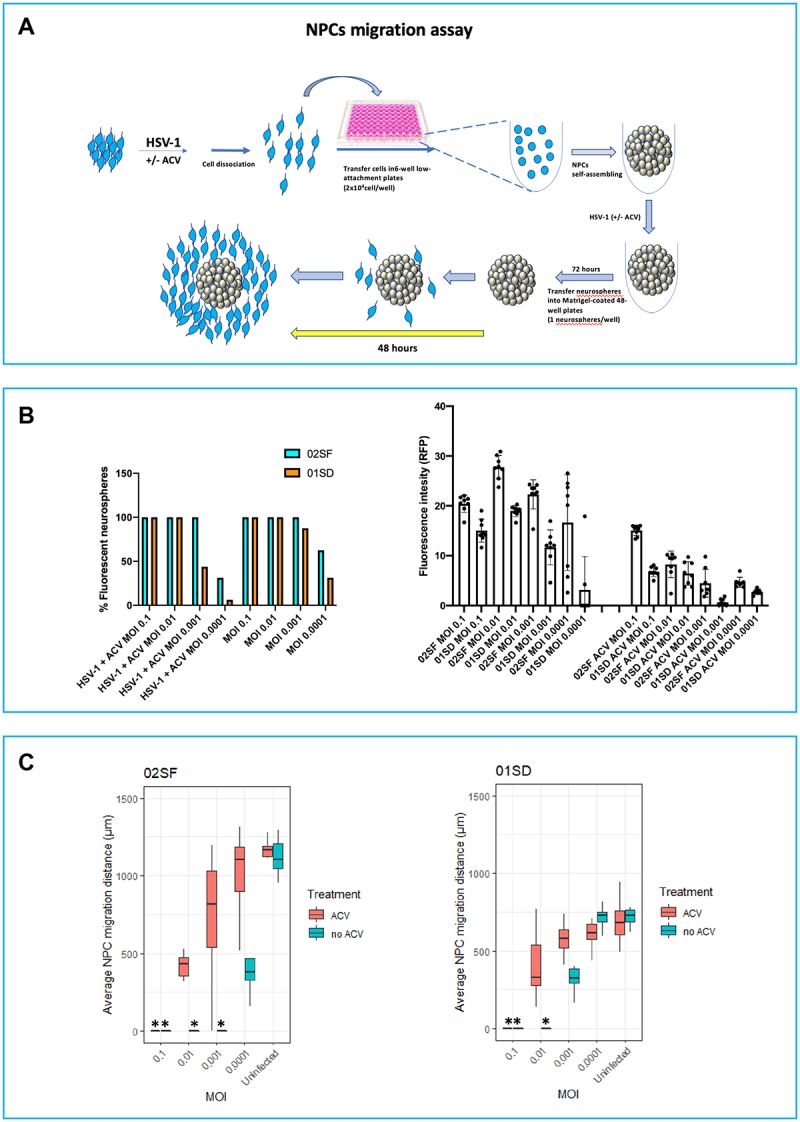
Variability in the effects of HSV-1 on NPCs migration. A: Schematic diagram of the neurosphere migration assay. 02SF and 01SD NPCs were infected at MOIs 0.1–0.0001 in the presence or absence of Acyclovir and cultured as neurospheres. Monolayer NPC cultures were dissociated and transferred into 96-well low attachment plates at the density of 2 × 10^4^ cells/well. In these culture conditions, one neurosphere/well was formed. Seventy-two hours later, neurospheres generated from uninfected and infected NPCs were transferred into Matrigel-coated 48-well plates (one neurosphere per well) to allow for the NPCs to migrate from the neurospheres. Sixteen neurospheres from each condition were analyzed. B: Analysis of the proportion of fluorescent neurospheres employed for the migration assay (Left) and EGFP fluorescent intensity (Right). C: NPCs migration was analyzed after 48 hours. The average migration of NPCs was calculated by measuring the distance moved of the five furthest cells from the edge of each neurosphere (greatest cellular migration). Error bars are standard deviations. The asterisks identify the cultures in which no cell migration from neurospheres occurred.

Next, we investigated whether HSV-1 has a differential effect on the migration of 02SF and 01SD NPCs. In the migration assay, it is important to utilize neurospheres of comparable size to reduce how size may influence outcome variability. Thus, we employed a different procedure to generate these 3D spherical NPCs aggregates, in order to reduce their variability in size (see Materials and Methods section; a summary of the scheme is shown in Figure 3a). After 72 hours, in the cultures infected at MOIs of 0.1–0.001, all the 02SF and 01SD neurospheres contained a high proportion of EGFP^+^-RFP^+^ cells. However, at an MOI of 0.0001, the proportion of fluorescent 01SD neurospheres was lower than 02SF (43.75% and 81.25% respectively) (Figure 3b). ACV treatment caused a 2.28-fold reduction in the proportion of the EGFP^+^-RFP^+^ neurospheres in 01SD cultures infected at MOI 0.001; however, it proved to be inefficacious in 02SF (Figure 3b). A striking difference in the proportion of EGFP^+^-RFP^+^ neurospheres between 02SF and 01SD neurospheres was observed in ACV-treated cultures infected at MOI 0.0001 (31.25% and 6.25%, respectively) (Figure 3b). At all MOIs, the RFP fluorescence intensity in infected 01SD cultures (in the presence or absence of ACV) was lower than 02SF (Figure 3b), indicating a difference in viral transcriptional activity between these two NPC lines.

Alt Text The panel A shows a schematic diagram of the neurosphere migration assay. The proportion of fluorescent neurospheres employed for the migration assay and EGFP fluorescent intensity is shown in Panel B. The panel C shows the analysis of the migration of neuronal progenitor cells from uninfected and infected neurospheres.

On day 3 p.i., uninfected and infected 02SF and 01SD neurospheres (16 neurospheres from each condition) were analyzed and transferred into Matrigel-coated 48-well plates (one neurosphere per well). Forty-eight hours later, the cellular migration of NPCs from each neurosphere was assessed (Figure 3a) (see Materials and Methods). In the absence of ACV, at an MOI of 0.001, no cell migration was observed from 02SF neurospheres, while NPCs migration was observed from 62.5% 01SD neurospheres. These results are consistent with a greater resistance of 01SD NPCs to infection than 02SF NPCs. At an MOI of 0.0001 NPCs could migrate from most of 02SF neurospheres. However, the distance they could migrate was significantly less than uninfected 02SF neurospheres. On the contrary, at an MOI of 0.0001 the distance migrated by 01SD NPCs was comparable to uninfected 01SD (Figure 3c), indicating that the virus did not cause migration abnormalities in 01SD NPCs at this MOI, as was observed in 02SF NPCs.

In the presence of ACV, HSV-1 also affected NPCs migration in a viral dose-dependent fashion, probably reflective of an ACV mediate inhibition of the modulating activity of HSV-1 on migration. However, the aberrant migration of 02SF NPCs caused by HSV-1 infection could be rescued by ACV only at MOI 0.0001. EGFP^+^-RFP^+^ cells from both 02SF and 01SD could migrate only a short distance (Figure 3c; Supplementary Figures 3 and 4).

In summary, these results indicate that HSV-1 impairs NPCs migration to a different extent in these two NPC lines. Similarly, the protective effect of the antiviral ACV on NPCs migration is viral dose-dependent. In fact, in both NPC lines, ACV treatment proved inefficacious at MOI 0.1 and modest at MOI 0.01. ACV effectively counteracted aberrant cell migration abnormalities only at MOI 0.0001.

## Discussion

A vast array of information concerning the interactions of HSV-1 with its hosts has been collected over time. *In vitro* modeling of the interaction of neurotropic viruses with human CNS has not been as feasible because of the difficulties isolating and expanding viable human CNS cells. In recent years, solutions to these limitations are provided by hiPSC technologies, which offer an unprecedented opportunity to generate large quantities of individual-specific disease-relevant CNS cells. However, the variability in the interaction of HSV-1 with specific cell types has been largely ignored and variability of the effects has not been investigated; prior studies have been limited to the modeling of rare single-gene inborn errors of immunity in hiPSC-derived CNS cells and human fibroblasts.^20–22^ Although it is well established that NPCs are highly susceptible to HSV-1, it is not known whether the virus can exert a differential impact on NPCs neurogenesis in different cell lines.^1,5,8^ Despite their fundamental importance in neurogenesis, there is a paucity of information regarding the consequence of HSV-1 infection of human NPCs on neurogenesis.^14,15,23,24^ An *in vitro* model has shown that HSV-1 (strain F) reduces the proliferation of mouse NPCs infected at MOI 0.5 for 24 hours.^14^ Furthermore, the quantification of mRNA expression of SOX2 (a transcription factor involved in cell proliferation) showed a significant downregulation.^25^ Similarly, a recent study reported a significant reduction in the transcription level of SOX2 in HSV-1 (strain F)-infected (MOIs 0.2 and 2) human monolayer NPC cultures at 24 hours p.i.^15^ However, the altered expression of this NPC marker was not observed by immunocytochemistry. In contrast, a decreased expression of SOX2 was detected in infected early stage (18-day old) organoids.^15^

In this study, we compared the effects of HSV-1 infection at low multiplicity of infection (MOIs 0.1–0.0001) on proliferation and migration of two hiPSC-derived NPCs derived from a healthy individual (02SF) and her first-degree relative (son, 01SD). Also, we investigated whether ACV therapy (which represents the gold standard for treating HSV infections) can ameliorate or revert to the control state, the infection-induced phenotypes. The effect of HSV-1 on NPCs proliferation was analyzed at days 2, 4, and 7 p.i. with neurosphere formation assay, which is a popular method to investigate the behavior of NPCs. The neurosphere diameters reflect the proliferative activity of NPCs.^19^ Overall, HSV-1 affected the proliferative activity of 02SF and 01SD in a viral dose-dependent fashion. The analysis of the percentage of fluorescent neurospheres at different time points following infection suggests a difference in the transcriptional activity of HSV-1 genes in 02SF and 02SF and 01SD NPCs (Figure 1, *middle panel*). This difference in transcriptional activity could be the result of an interindividual difference in the efficiency of silencing of HSV-1 lytic genes during latency. It is known that heterochromatic silencing of HSV-1 during latency varies between HSV-1 strains,^26^ and the results presented here may suggest that host factors play a role in this silencing as well. This possibility has broader implications in that it could be a factor in not only why some individuals reactivate HSV more efficiently but also why HSV-1 infections in the CNS may have pathological consequences for some individuals and not others, including the progression of Alzheimer’s Disease. The ACV treatment could efficiently counteract the inhibitory action of the virus on NPCs proliferation in 02SF but not in 01SD, where only the neurospheres derived from NPCs infected at MOI 0.0001 showed sizes comparable to those derived from uninfected neurospheres (Figure 1, *bottom panel*). These results show differences in the efficacy of ACV to protect 02SF and 01SD NPCs functions from the effects of HSV-1 infections.

We have previously shown that HSV-1 impairs NPCs migration in a viral dose-dependent fashion.^8^ In this study, we enquired whether HSV-1 can impair, to a different extent, the migration of NPCs from different individuals. The 01SD NPCs migration was reduced when compared to 02SF NPCs. A decreased NPCs migration represents a phenotype described in schizophrenia subjects.^27^ Although HSV-1 affected NPCs migration in a viral dose-dependent fashion in both lines, HSV-1 impacted 01SD NPCs migration to a lesser extent than it did to 02SF at MOIs 0.001–0.0001 (Figure 3c). This difference may reflect the presence of a lower proportion of infected cells in 01SD neurospheres when compared to 02SF. ACV treatment proved more efficacious in infected 01SD NPCs than 02SF NPCs during the preparation of neurospheres for the migration assay (Figure 3b). However, the higher sensitivity of 01SD NPCs to ACV did not lead to an improved rescue of the migration abnormality of NPCs migration when compared to 02SF NPCs.

Together, these results pose the question of whether the different impact that HSV-1 may have on the migration of NPCs in different individuals contributes to the heterogeneity of cognitive sequelae across encephalitis survivors.

It should be noted the difference in the EGFP^+^-RFP^+^ fractions of the neurospheres generated for the neurosphere assay and the NPC migration assay. This difference relies on the fact that neurospheres generated for the formation assay represent an expansion of a few initial infected and/or uninfected cells, while neurospheres generated for the migration assay are the product of the self-assembling of the 2 × 10^4^ cells infected at different MOIs.

The difference in structural stability between the infected 02SF and 01SD neurospheres (Figure 1 and Supplementary Figure 1) indicates that the extracellular matrix degradation and/or remodeling is affected more acutely in the former. These results indicate a variability in the degree of the HSV-1 pathogenicity among individuals in the absence of immune cells.

The difference in the sensitivity to ACV observed between 02SF and 01SD NPCs may stem from host-related differences in ACV metabolism (differential expression of one of some of the seven kinases involved in the second and third rounds of ACV phosphorylation or other mechanisms that prevent them from achieving the minimal acyclo-GTP concentration required in the infected cells to inhibit viral replication). Also, differences in antiviral responses to ACV may be observed due to natural variation in baseline susceptibility to HSV-1 infection, which is ascribed to differential basal-level expression of components of innate immune signaling pathways. We hypothesized that either one or both mechanisms underlie the difference in sensitivity to ACV observed between the two NPC lines.

The limited number of hiPSC lines employed in this study is an important caveat. Although the quality controls performed on 02SF and 01SD hiPSC lines did not indicate the presence of evident genomic rearrangement, we cannot exclude the possibility that the difference described between these two lines may reflect abnormalities during the dedifferentiation of fibroblasts or redifferentiation of hiPSCs. Further studies involving multiple hiPSC lines are needed to investigate whether the differences observed between 02SF and −01SD reflect a more general phenomenon of interindividual variability in the impact of HSV-1 on NPCs neurogenesis, and the extent of variation in these phenotypes that may exist in the population. The consensus in the stem cells biology field is to employ hiPSC lines from different individuals rather multiple clones generated from the same individuals when performing comparisons between groups of subjects.^28^

Additional studies are needed to investigate whether underlying differences in proliferation (Supplementary Figure 2) contribute to the difference in the impact that HSV-1 infection has on self-renewal and migration observed between these two NPC lines.

In summary, our results raise the notion that sensitivity to ACV and the impact that HSV-1 has on neurogenesis are subject to interindividual differences.

## Supplementary Material

Supplemental Material
