# Supplementary figures and images for: Variations in Aspects of Neural Precursor Cell Neurogenesis in a Human Model of HSV-1 Infection
Source: Organogenesis. 2022 Apr 6;18(1):2055354. doi: 10.1080/15476278.2022.2055354 (PMC8993067; doi:10.1080/15476278.2022.2055354)

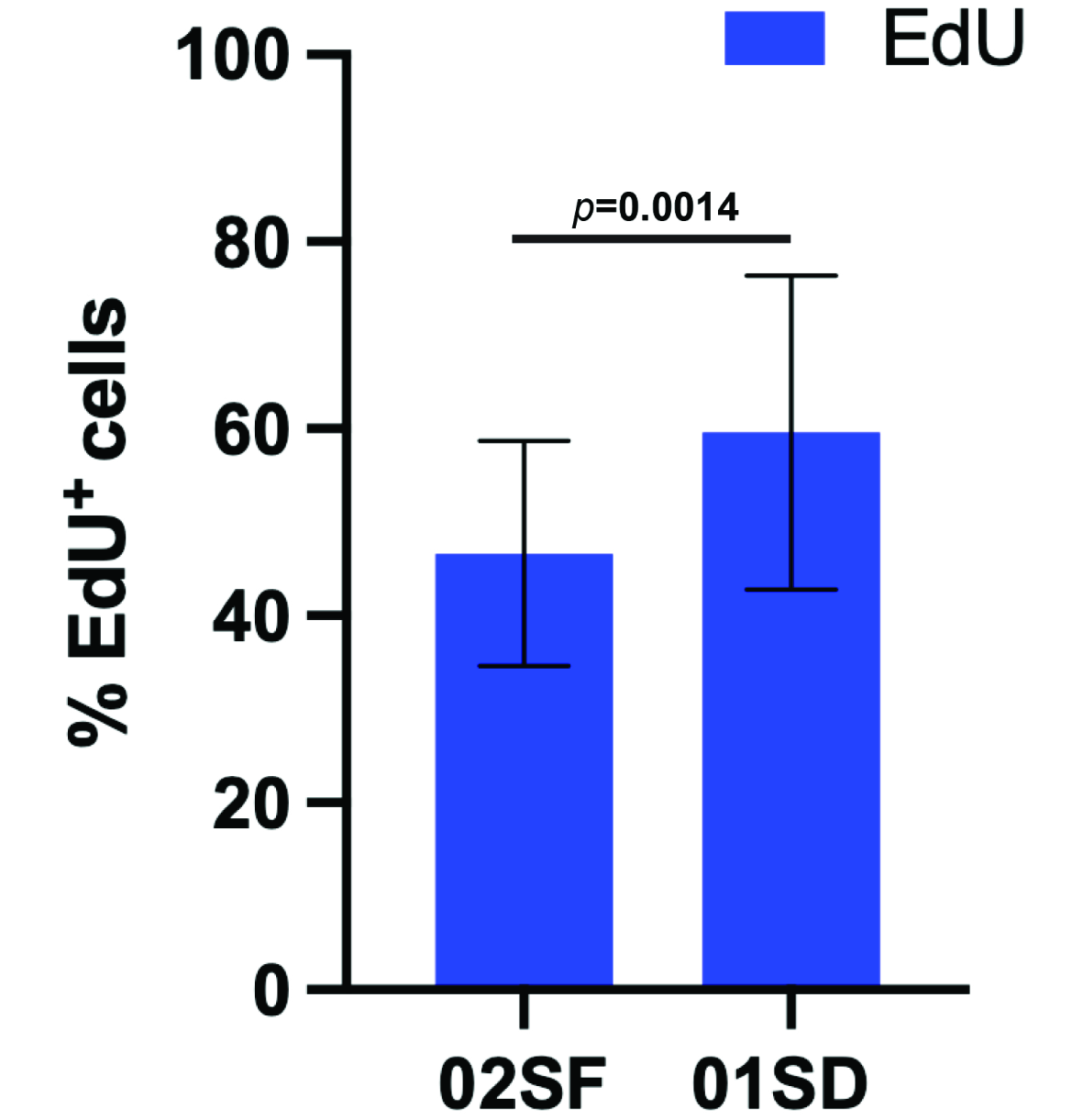

Supplement: Supplemental Material [file KOGG_A_2055354_SM5676.zip › Supplementary Fig 2.tif]

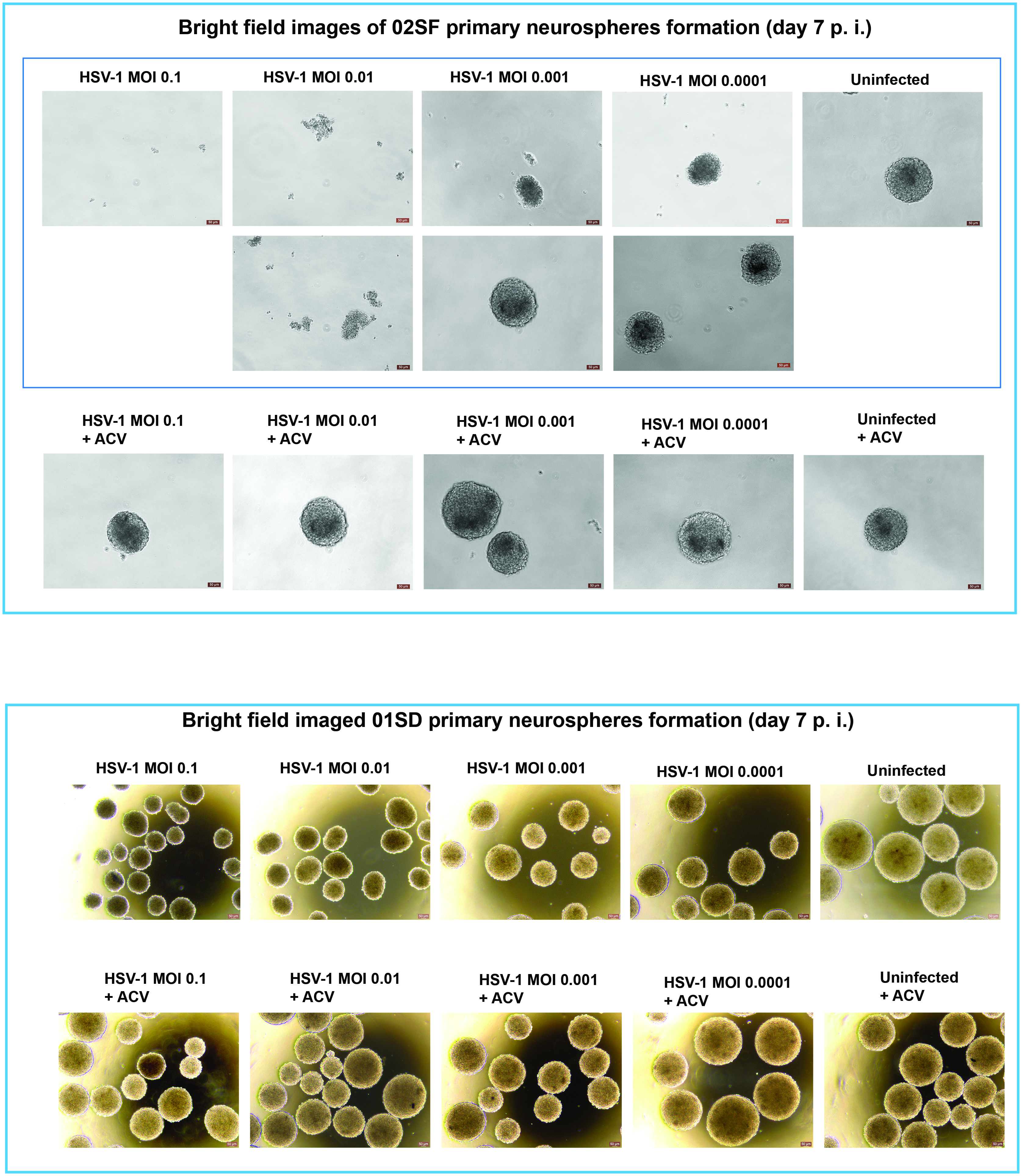

Supplement: Supplemental Material [file KOGG_A_2055354_SM5676.zip › Supplementary Figure 1 rev.tif]
